# DNA Strand Breaks at Centromeres: Friend or Foe?

**DOI:** 10.1016/j.semcdb.2023.10.004

**Published:** 2023-10-21

**Authors:** Emily Graham, Fumiko Esashi

**Affiliations:** 1 Sir William Dunn School of Pathology, University of Oxford, Oxford, UK

**Keywords:** Centromeres, DNA Damage, DNA Repair, Cell Cycle, Quiescence

## Abstract

Centromeres are large structural regions in the genomic DNA, which are essential for accurately transmitting a complete set of chromosomes to daughter cells during cell division. In humans, centromeres consist of highly repetitive α-satellite DNA sequences and unique epigenetic components, forming large proteinaceous structures required for chromosome segregation. Despite their biological importance, there is a growing body of evidence for centromere breakage across the cell cycle, including periods of quiescence. In this review, we provide an up-to-date examination of the distinct centromere environments at different stages of the cell cycle, highlighting their plausible contribution to centromere breakage. Additionally, we explore the implications of these breaks on centromere function, both in terms of negative consequences and potential positive effects.

## Introduction

Centromeres provide the location of kinetochore nucleation and microtubule attachment and are therefore essential for accurate chromosome transmission. Centromere instability and the resultant mitotic defects are common hallmarks of human disease, including tumourigenesis [1]–[3], increased understanding of which has exciting implications for cancer therapy (recently reviewed in [4]). Hence, there is no denying that the maintenance of centromere functionality holds fundamental importance for our health. Paradoxically, however, there is growing recognition that centromeres are common hotspots of DNA breaks. The emerging question is why, from a physiological perspective, DNA breaks at centromeres arise so frequently. Are they randomly induced and toxic, or are these breaks introduced in a controlled fashion for a functional purpose? In this review, we aim to untangle the mechanisms underlying centromeric DNA breaks and their biological significance. We will firstly outline the overall composition of human centromeres, and then provide an updated overview of their distinct structure at each cell cycle stage, the plausible source of DNA breaks and the mechanisms repairing these breaks. By doing so, we hope to shed a new perspective on this somewhat peculiar characteristic of centromeric DNA in humans.

### 1 Centromere structure

**Figure 1:**
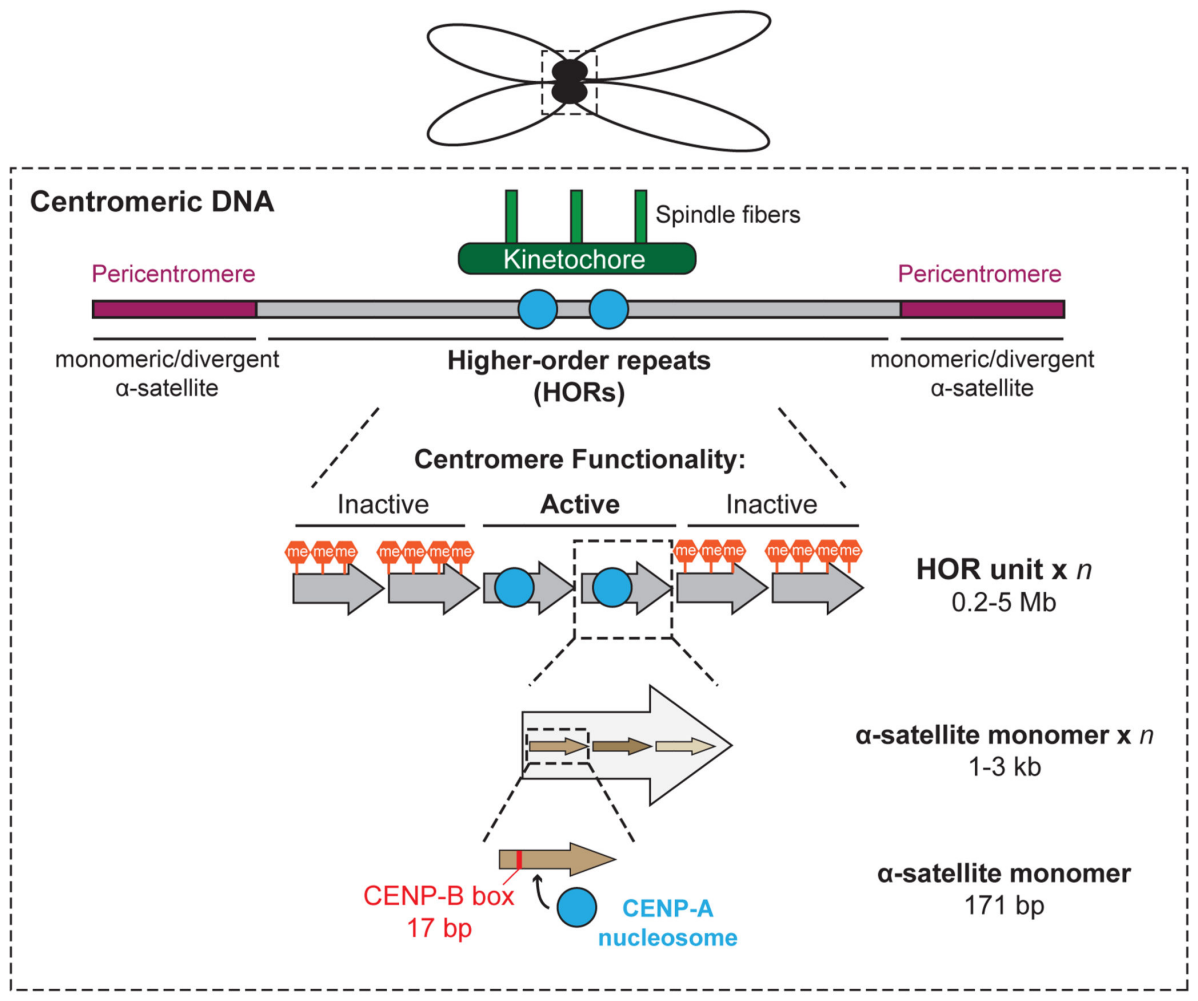
Centromere structure Centromeres form the site of kinetochore attachment and consist of repetitive α-satellite monomers arranged in a head-to-tail tandem fashion (brown arrows), organised into 1 - 3 kb higher order repeats (HORs) (large grey arrows). These HORs are further repeated to span for approximately 0.2 - 5 Mb, which are flanked by non-HOR pericentromeric regions consist of monomeric or divergent α-satelite sequences. The functionally active centromere is defined epigenetically with CENP-A-containing nucleosomes (blue circles), while those inactive HORs are enriched with methylated cytosines (orange hexagons). 171 bp α-satellite monomers also contain a 17 bp motif termed the CENP-B box, the site of CENP-B binding (red line).

Human centromeric DNA comprises tandem repeats of AT-rich, α-satellite monomers that are 171 base pairs long and share 50-80% homology. In each chromosome, a defined number of α-satellite monomers are arranged in a head-to-tail orientation, creating a larger higher-order repeat (HOR) unit, which is further repeated for several megabases to form the core regions of centromeres [2], [5] (Figure 1). Extending from the core centromere, pericentromeric DNA consists of α-satellite monomers and other satellite DNA stretches of varying sequences and length [1], [6]. Despite this unique DNA nature, functionally active centromeres are specified epigenetically through the presence of the histone H3 variant, CENP-A, which occupies small sections of HORs [7]–[9].

CENP-A loading is temporally and spatially regulated during the cell cycle in growing cells [1], [10]. Guided by the preexisting CENP-A from the previous cell cycle, new CENP-A is incorporated into centromeres in late mitosis and early G1 [10], [11] by a histone chaperone Holliday junction recognition protein, HJURP [6], [12]–[23]. Other centromere-associated proteins, i.e. CENP-B, CENP-C, and CENP-I, are also implicated in CENP-A deposition, albeit to a lesser degree compared to HJURP [24]–[31]. In the absence of preexisting CENP-A, however, CENP-B, which binds 17 base pair CENP-B boxes present throughout the α-satellite HORs (Figure 1) on all chromosomes except the Y chromosome, plays an essential role for depositing CENP-A *de novo* at centromeres [32], [33].

While centromeres and pericentromeres have been considered largely heterochromatic and transcriptionally silent [34], human and fly HORs were found to contain transcriptionally permissive markers, H3K4me2 and H3K36me2 [34]–[36]. Supporting this observation, it has become evident that, across several different organisms, centromeres are actively transcribed by RNA Polymerase II (RNAPII) [36]–[42], though at different stages of the cell cycle, i.e. mitosis and early G1 in humans, mitosis in flies, and S in mice and yeasts [43]. In humans, loss of centromeric transcription reduces CENP-A levels [38], highlighting the importance of this event for centromere specification. At least two mechanisms have been proposed for how centromere transcription influences CENP-A deposition. Firstly, centromeric long non-coding RNA (lncRNA) associate with HJURP to form nucleoprotein complexes, which in turn assist direct loading of CENP-A to centromeres [37]. However, another study using single molecule FISH (smFISH) analysis revealed that, while α-satellite transcripts are detectable throughout the cell cycle, they are spatially separated from their centromere of origin [44], arguing against this model. The second model proposes that the process of centromere transcription, rather than the presence of α-satellite RNA molecules themselves, is important for CENP-A deposition by opening the centromeric chromatin [39], [44].

Once incorporated into centromeric chromatin, CENP-A fosters the assembly of the sixteen-subunit constitutive centromere-associated network (CCAN) in G1 [1], [45] which then dynamically recruits the components of the outer kinetochore KNL1-MIS12-NDC80 (KMN) complex during S and G2 [46], [47]. In early mitosis, the centromere-associated KMN complex is stabilised and captured by mitotic spindle fibres, the fulfilment of which is monitored by the spindle assembly checkpoint (SAC) to ensure accurate chromosome segregation [1], [5], [17].

## 2 Centromere integrity throughout the cell cycle

DNA fragility at and around centromeres has been long observed through chromosome pathology [2]. Recent advancements in technologies detecting DNA strand breaks by next-generation sequencing (NGS) [48], accompanied with the completion of the first human reference genome assembly including centromeric regions [49], have uncovered DNA strand breaks explicitly within HORs in humans [50], [51]. Breaks in HORs, either single- or double-stranded, are alternatively detected microscopically, by combining exonuclease III treatment followed by fluorescence *in situ* hybridization (exo-FISH) [51] (further discussed in section 2.4.1). Whilst there are some technical drawbacks for each methodology on its own (discussed in [51]), these analyses collectively support the notion that centromeres are common hotspots of DNA breaks [51]. This reconciles, at least in part, the recognition that centromere DNA sequences diverge significantly between species, and even between individuals [52].

To fully appreciate the impact of centromeric DNA breaks in both growing and resting conditions, in the following sections, we examine plausible sources of centromere fragility in the context of normal physiology across the cell cycle, and discuss the potential cost and benefit of these events at centromeres.

### 2.1 Centromeres in G1

G1 is the phase after cell division when cells are prepared for the forthcoming phase of DNA replication. Chromosomes are largely free from the outer kinetochore and the condensin complexes that are required for chromosome segregation. Instead, transcription resumes to produce factors required for DNA replication, and epigenetic components, including those at centromeres, are reinstated.

**Figure 2:**
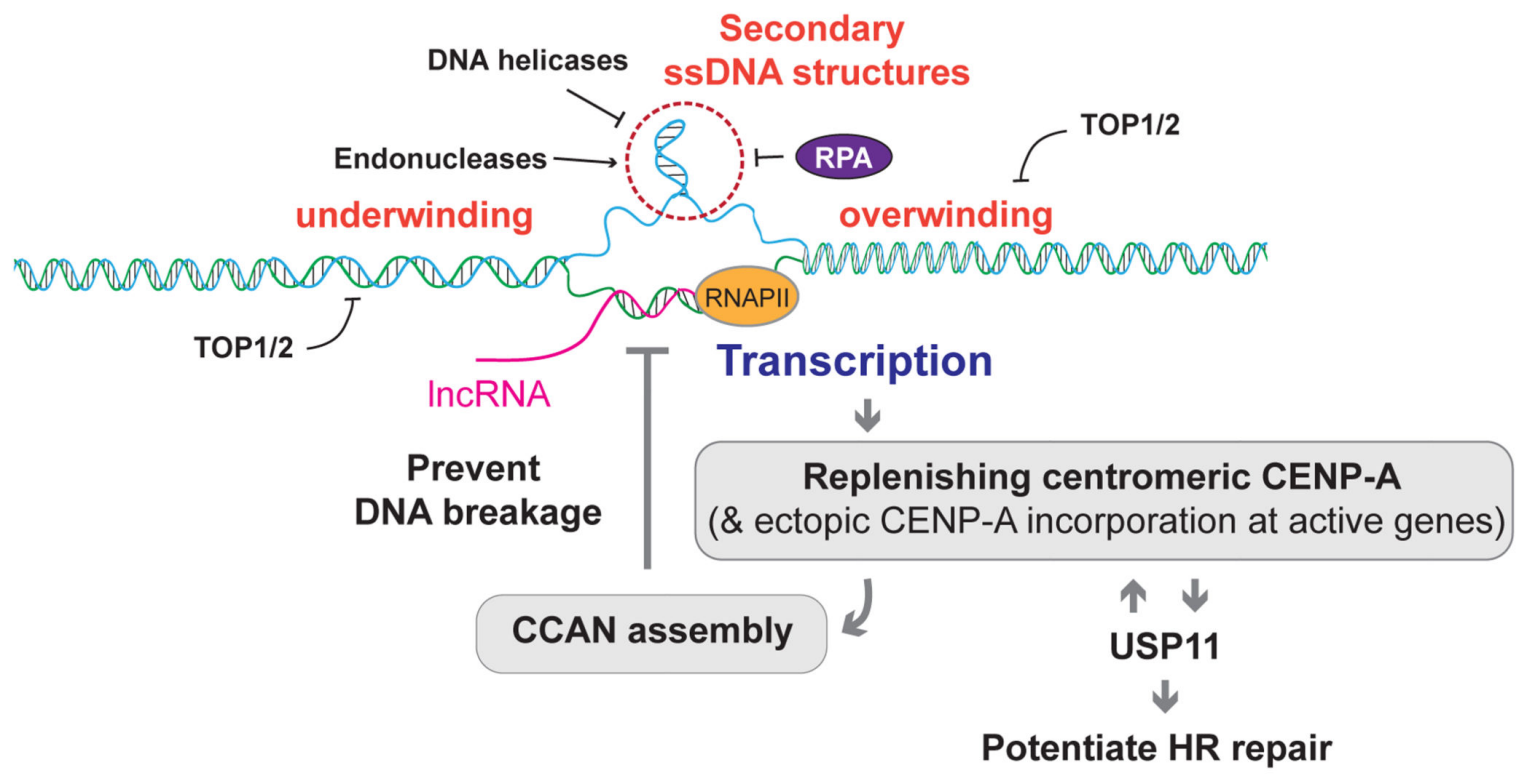
Events at centromeres in G1 phase Centromeres are subject to RNA polymerase II (RNAPII)-mediated transcription, which is important for replenishing centromeric CENP-A. Accompanying DNA unwinding, torsional stress and RNA/DNA hybrid (R-loop) formation could trigger the creation of secondary DNA structures, underlying the induction of DNA breaks, conceivably by endogenous structure-specific endonucleases. Detrimental DNA breaks can be alleviated by DNA helicases and topoisomerases (TOP1/2) targeting the secondary DNA structures. DNA fragility can also be alleviated by the constitutive centromere associated network (CCAN), which, once established, down-regulates centromeric transcription. DNA breaks in G1 can also be repaired by homologous recombination, enabled by the ubiquitin specific peptidase 11 (USP11) which also supports CENP-A replenishment.

Transcription involves the unwinding of double stranded DNA (dsDNA), therefore inevitably introducing transient bubbles and torsional stress. If left unresolved, this process can provide an opportunity for displaced single stranded DNA (ssDNA) to form secondary structures, such as stem-loops, hairpins, cruciform, and i-motifs (Figure 2) [53]. Such structures have all been associated with centromeres in humans, flies, and yeasts [53]–[58]. Interestingly, analysis of centromere DNA sequences across species revealed enrichment of dyad symmetries, which can adopt these structures [59], [60]. This correlates with the absence of CENP-B in these organisms, leading to the interesting hypothesis that such structures may have equivalent roles to CENP-B, such as *de novo* CENP-A deposition. Transcription-associated torsional stress can also lead centromeric RNA to form hybrids with template DNA, named R-loops, which, if left unresolved, may further stabilise the secondary structures of displaced ssDNA [61]–[63].

Structure-forming DNA and R-loops are likely resolved efficiently by the ssDNA binding replication protein A (RPA) [64] or by enzymes reported to act in the context of DNA replication (discussed further in section 2.2). Alternatively, type I or type II topoisomerases, which introduce transient single-strand breaks (SSBs) or double-strand breaks (DSBs), respectively, can alleviate the formation of such structures by relieving local torsional stress [65]. Among those, topoisomerase IIα (TOP2A) is probably best known to act at centromeres. TOP2A also displays biochemical activity to cleave secondary hairpin DNA structures in human α-satellite sequences *in vitro* [58], although the exact position of cleavage in a biological context remains contested [58], [66]–[70]. These secondary DNA structures can also be targeted by other structure specific endonucleases, such as MUS81 and SLX4, although these enzymes are largely down-regulated during G1 [71].

#### 2.1.1 Repair of centromeric DNA breaks in G1

It is not yet known whether centromeres are subject to intrinsic DNA breaks which are *de novo* induced in G1. Regardless, there are several studies addressing the repair of exogenously induced centromeric DNA breaks in G1. A close inspection of cells exposed to ionising radiation suggested that the heterochromatic nature of centromere DNA restricts the mobility of break ends, limiting incorrect DSB repair which can result in gross chromosomal rearrangements (GCRs) [72]. A more recent study using an RNA-guided nuclease Cas9 similarly found that centromeric DSBs do not cause chromosomal translocations, proposing the involvement of homologous recombination (HR) in repairing DNA breaks *in cis* [73]. They observed the recruitment of RAD51, a key enzyme catalysing HR, to centromeres upon the Cas9-mediated induction of centromeric DNA breaks, which are enriched with active transcription chromatin marker, H3K4me2, and R-loops. While the impact of R-loops in aiding or impeding HR-mediated repair remains debated [74], Yilmaz *et al*. found that the enrichment of RAD51 is dependent on Ubiquitin Specific Peptidase 11 (USP11) which is implicated in regulating R-loops [75] and HR [76]. Canonically, RAD51 is recruited to broken DNA through the HR mediator complex comprising breast cancer 2 (BRCA2), partner and localizer of BRCA2 (PALB2), and breast cancer 1 (BRCA1). USP11 was previously shown to deubiquitylate PALB2 during S and G2 to promote its interaction with BRCA1, hence promoting HR in these phases of the cell cycle [76]. Yilmaz *et al*. proposes that USP11 acts locally at centromeres during G1 to promote HR, while it also facilitates CENP-A deposition by deubiquitylating the CENP-A chaperone, HJURP.

Together, it seems conceivable that there is a functional connection between centromeric DNA breaks, HR repair, and CENP-A deposition in G1 [73]. HR is typically considered inactive during G1 partly due to the lack of the homologous sister chromatid repair template. However, the repetitive nature of centromere α-satellite DNA sequences may negate this requirement through intra-strand invasion of centromere repeats on the same chromatid (discussed further in section 2.3.4) [72], [77]. Notably, HJURP itself was first identified to interact with repair factors, MSH5 and NBS1, as part of the HR repair pathway, forming foci in response to DNA damage in S phase and binding Holliday junctions *in vitro* [78]. Further, UV laser stripe experiments and I-*Sce*I endonuclease-induced DSBs in human and mouse cells revealed rapid recruitment of CENP-A to sites of damage, along with CCAN constituents CENP-N, CENP-T, and CENP-U [79]. The exact function of CENP-A recruitment at DNA lesions is still unclear but could implicate a role for DNA breaks in centromere specification and CENP-A deposition.

### 2.2 Centromeres in S/G2

**Figure 3:**
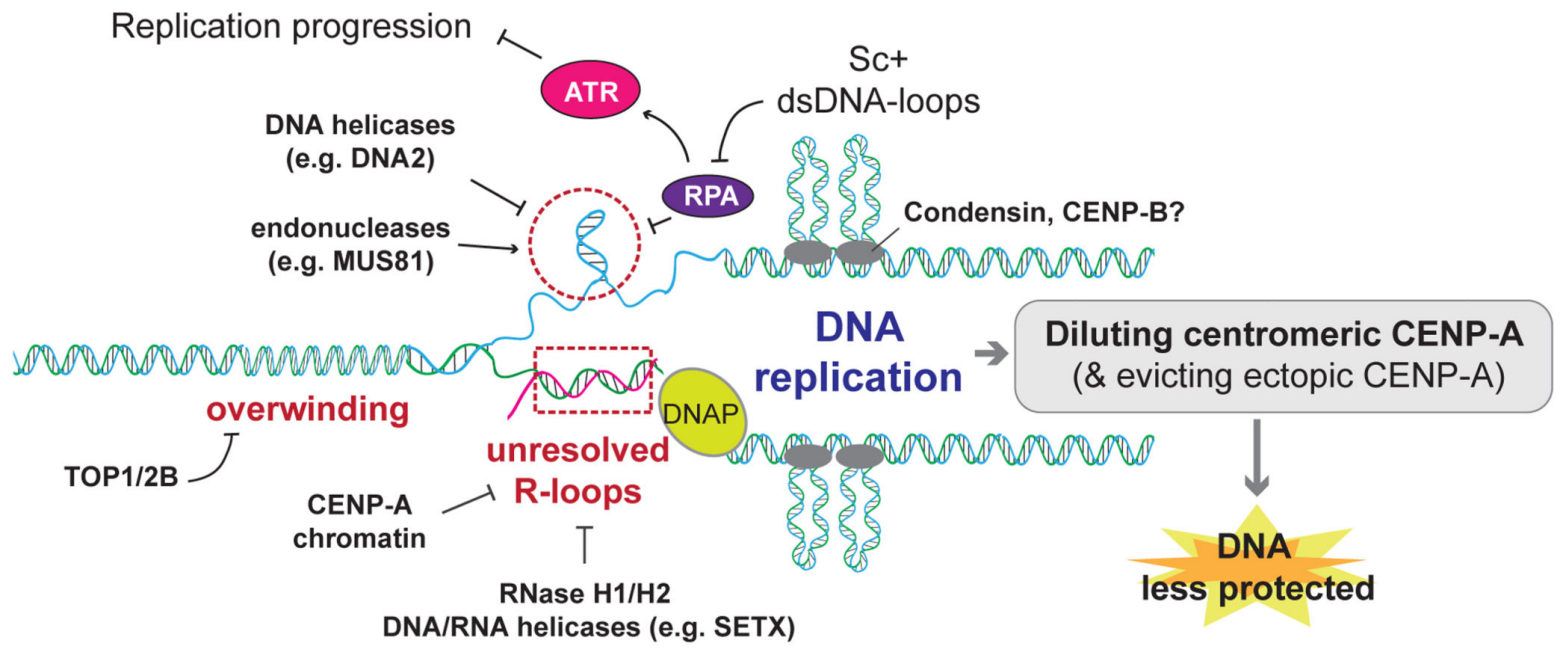
Events at centromeres in S and G2 phases Centromeres undergo replication in late S-phase, and accompanying DNA unwinding, torsional stress and unresolved RNA/DNA hybrids (R-loops) could underlie the induction of DNA breaks. Replication protein A (RPA) binding to replication forks is impeded due to supercoiled DNA loops generated behind the fork. In this way, ATR (Ataxia telangiectasia and Rad3-related) remains inactive and replication can proceed through the centromeric DNA, while leaving these regions under-protected. CENP-A, which alleviates DNA fragility, is also diluted during replication. DNA breakage can be alleviated by DNA helicases, topoisomerases, RNase H, and DNA/RNA helicases. DNAP denotes DNA polymerase. Sc+ denotes positively supercoiled dsDNA.

Like transcription, DNA replication entails dsDNA unwinding, introducing torsional stress, hence providing the opportunity for extended ssDNA at replication forks to form secondary DNA structures (Figure 3). At centromeres, which are replicated late in humans [80], no new loading of CENP-A takes place during S and G2 phase and the CENP-A containing nucleosomes are simply diluted evenly between sister chromatids [81]. Therefore, it seems unlikely that the presence of secondary DNA structures on its own promotes CENP-A loading. Rather, ectopically incorporated CENP-A at actively transcribed genes on chromosome arms is removed, hence replication works as a proof-reading process to ensure correct localisation of CENP-A [82]. Nevertheless, centromeres are recognised as challenging regions of the genome for DNA replication. Structure-forming DNA is understood to cause uncoupling of DNA replication, which predisposes centromeres to DNA instability and rearrangement [2]. Such characteristics are exacerbated when cells are exposed to replication stress, such as those induced by an inhibitor of B-family DNA polymerases, aphidicolin, and in cancer cells which commonly bear intrinsic replication stress [50], [51], [54]. Below, some distinctive features of centromeric DNA during replication are highlighted.

#### 2.2.1 Secondary DNA structures and topologically generated loops at replication forks

Structure-forming DNA can impede replication fork progression leading to the suggestion that centromeres are inherently difficult-to-replicate [3], [54], [83], [84]. However, *in vitro* replication reconstitution of bacterial artificial chromosomes (BACs) containing human α-satellite DNA sequences using frogs’ egg extract found no altered replication kinetics in untreated conditions compared to other chromosome regions [54], suggesting that centromere replication is relatively unperturbed under physiological conditions. Intriguingly, an examination using electron microscopy (EM) further revealed replication- and condensin-dependent dsDNA loops which are positively supercoiled and accumulate behind the replication fork (Figure 3) [54]. These supercoiled DNA loops limit the accumulation of RPA at replication forks and, in this way, restrict the local activation of the replication checkpoint kinase, ATR. This mechanism is proposed to ensure timely completion of DNA replication at centromeres during S phase. Of note, a recent study suggests a DNA damage-independent role for ATR in maintaining centromeric CENP-A occupancy [85]. These observations, along with another centromeric role of ATR in mitosis (discussed further in section 2.3.2), imply an elaborate mechanism by which ATR affects centromeres, warranting further investigation.

Despite the seemingly attenuated activity of ATR at centromeres in S and G2 phases, numerous DNA repair factors, including those involved in mismatch repair (MSH2, MSH6), base excision repair (XRCC1, PARP1) and DSB repair (XRCC5, MRE11, RAD50) are found enriched at these regions, indicating their distinctive requirement in maintaining centromere stability during DNA replication [54], [86]. Along the same line, the DNA replication helicase/nuclease 2 (DNA2) was also found to localise to centromeric regions [87]. DNA2 possesses helicase and nuclease enzymatic activities, both of which are required to resolve DNA-dependent obstacles, such as hairpins and stem-loops, *in vitro*. This property of DNA2 is proposed to promote efficient replication, while preventing the accumulation and activation of ATR at centromeres. Failure of DNA2 to resolve centromeric DNA leads to impaired centromeric DNA replication and CENP-A deposition, and mitotic defects [87].

As in the case for transcription, discussed in section 2.1, TOP2 also plays a key role in resolving topologically generated loops. From late S phase to anaphase, TOP2 acts with the structural maintenance of chromosomes (SMC) complexes, such as condensin (composed of SMC2/4) and cohesin (composed of SMC1/3) [88], at active centromeric chromatin to facilitate architectural changes of chromatin [65], [89]–[92]. In budding yeast, TOP2 is also shown to act with the third SMC5/6-based complex to resolve replication-associated topological stress which would otherwise induce replicative DNA breakage [93]. A role for the SMC5/6 complex in chromatin dynamics at human centromeres is similarly reported [94].

Overall, secondary DNA structures and topologically generated loops at centromeres may create an unusual requirement for a multitude of factors, including checkpoint regulators, repair factors and chromatin architecture components, to enable efficient DNA replication while alleviating centromere instability.

#### 2.2.2 Resolution of R-loops and conflicting transcription

Centromeric transcription, which has a positive role in G1 for loading CENP-A, could become an enemy during DNA replication. While centromeric transcription is largely limited to mitosis and G1 phase in human cells, untimely transcription or unresolved R-loops in S phase can impede DNA replication, resulting in centromere instability (Figure 3) [95]–[98]. Hence, it would not be too surprising that the process of centromeric transcription is tightly regulated during the cell cycle. Indeed, CENP-C was found to suppress centromeric transcription, suggesting that once CENP-C is recruited by CENP-A to centromeres, transcription could become attenuated (Figure 2) [44], [99]. Corroborating this notion, CENP-A itself was shown to suppress the level of R-loops in late S phase to facilitate efficient DNA replication. However, the dilution of CENP-A between sister chromatids may lead to a transient increase in vulnerability if untimely transcription persists (Figure 3) [96].

Alleviating this associated vulnerability at centromeres, a component of the HR mediator complex, BRCA1, was shown to accumulate at centromeres in an R-loop dependent manner, where it conversely offsets R-loop accumulation by recruiting the RNA/DNA helicase senataxin (SETX), and in this way, promotes the completion of replication at centromeres [97]. While incomplete centromere replication, such as those seen upon the depletion of CENP-A or BRCA1, can be resolved through mitotic DNA synthesis (MiDAS) (discussed further in section 2.3.4), such events can contribute to centromere fragility due to aberrant chromosome fusions, sister chromatid exchanges (SCE) and loss of heterozygosity [96], [97].

Curiously, while unresolved R-loops at centromeres appear toxic for DNA replication, there is evidence supporting the beneficial role of centromeric transcripts in S phase. Namely, centromeric transcripts underlie sister chromatid cohesion, which is established in S phase and lasts until mitosis [99]. Tethering the transcriptional suppressor KOX1 to the centromere decreased centromeric transcripts and cohesion leading to mitotic progression defects. Therefore, it seems that centromere transcription is a dynamically regulated process required for centromere function but detrimental for centromere stability if dysregulated.

#### 2.2.3 Repair of centromeric DNA breaks in S/G2

**Figure 4:**
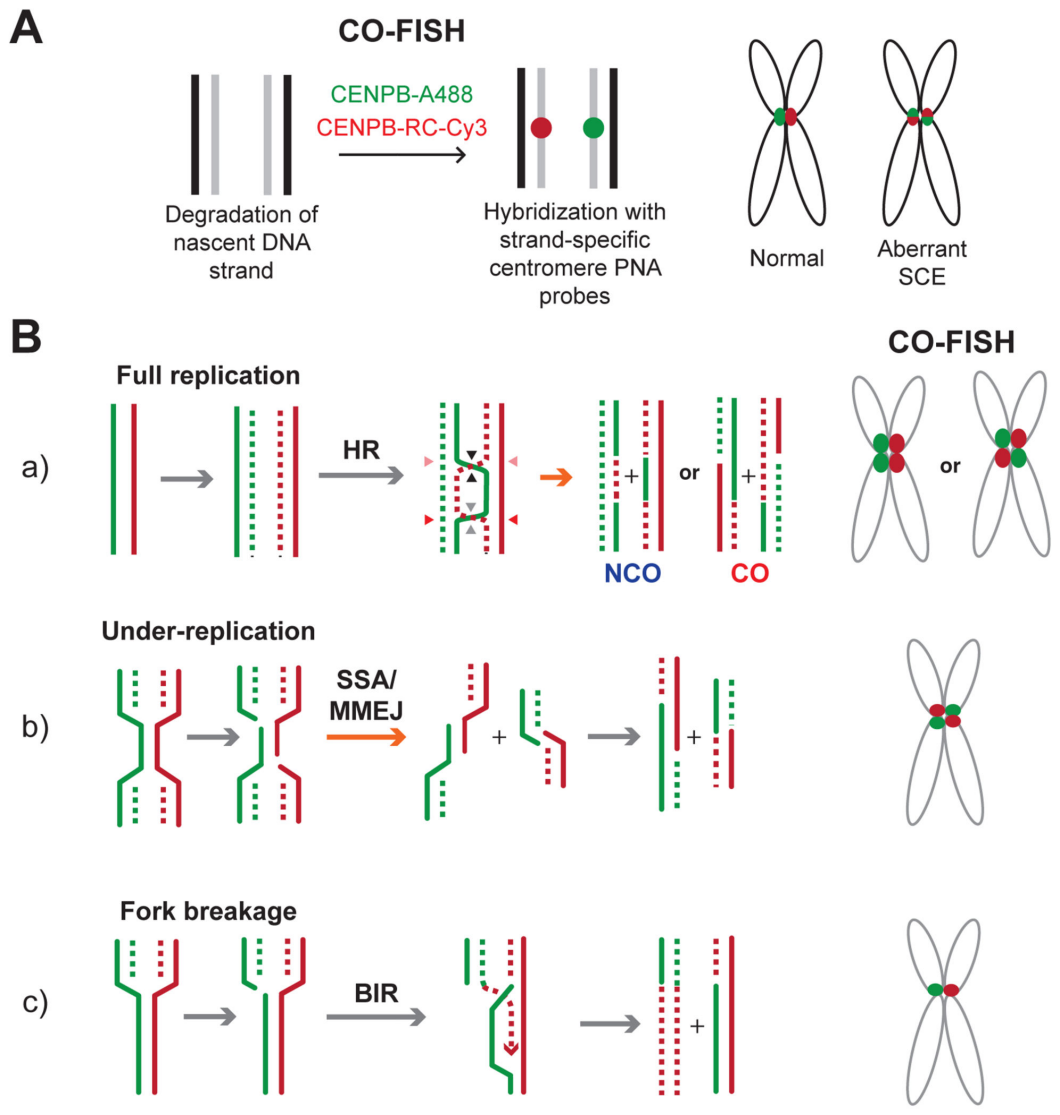
Replication-associated DNA break repair at centromeres **A**. Chromosome orientation fluorescence in situ hybridization (CO-FISH) involves labelling sister chromatids with strand-specific centromere-specific probes following digestion of nascent DNA. Distortion of FISH signals can be defined as aberrant sister chromatid exchange (SCE). **B**. Prospective replication-associated DNA repair events at centromeres. (a) Homologous recombination (HR) between sister chromatids can be resolved either in non-crossover mode (NCO) or cross-over mode (CO). Only CO mode of HR is detectable by CO-FISH. (b) Under-replicated DNA can be repaired by single strand annealing (SSA) or microhomology-mediated end joining (MMEJ), resulting in aberrant CO-FISH signals. (c) Fork breakage can induce break-induced replication (BIR), which is hard to detect by CO-FISH.

The combination of replication-associated breaks and the highly repetitive nature of centromeres makes this region highly susceptible to recombination during S phase when HR repair is canonically active (Figure 4). However, there is ongoing debate regarding the impact of centromeric recombination: whether it disrupts centromere integrity and functionality [2], [100], [101] or conversely serves a functional benefit, facilitating the evolution of better functioning centromere DNA sequences [51], [52].

The notion supporting the harmful impact of centromeric recombination is initially founded on studies demonstrating that centromeres are recombination cold spots during eukaryotic meiosis [102], [103] reasoning that centromeric recombination has a negative effect on meiotic chromosome segregation, breakage, and loss [104], [105]. Accordingly, centromeric recombination in somatic cells has attracted growing interest in recent years, revealing frequent SCEs, which are detected by strand-specific chromosome orientation fluorescence *in situ* hybridization (CO-FISH). It is important to consider that, while SCEs could infer crossover resolution of HR, they also reflect the outcomes of multiple error-prone repair mechanisms, such as single-strand annealing (SSA), break-induced replication (BIR) and microhomology mediated end-joining (MMEJ) (Figure 4) [73], [96], [106]–[109]. Further, in somatic cells, HR events are predominantly resolved in non-crossover mode, including synthesis-dependent strand annealing (SDSA) or the dissolution of Holliday junctions by the dissolvasome complex consisting of topoisomerase IIIα (TOP3A) and BLM helicase [110]. These non-crossover events cannot be detected by CO-FISH, suggesting that recombination events remain largely undetected at centromeres [111]. Also, BIR is difficult to detect by CO-FISH, as this technique digests the nascent DNA strand to observe exchanges between the parental strands. Hence, centromeric SCEs, detectable by CO-FISH, should be interpreted cautiously, although can be used as an indicator of error-prone repair outcomes.

The propensity for centromere SCEs, to a large extent, appears to reflect DNA replication stress. DNA lesions resulting from replication stress can promote DNA rearrangements through erroneous repair pathways such as SSA and MMEJ. Such events could theoretically occur frequently at highly repetitive centromeric regions, leading to the loss of HORs and CENP-A binding sites, and hence negatively impacting centromere stability [100]. This notion is supported by the observation that cancer cell lines display reduced chromatin-bound CENP-A and ~15% increase in centromeric SCEs compared to non-cancerous RPE1 cells [112], [113].

Several mechanisms limiting error-prone repair of centromeric DNA breaks have been proposed. Given that large parts of centromeres maintain their heterochromatic status through CpG methylation (Figure 1), this epigenetic marking was suggested to suppress erroneous recombination within α-satellite arrays [100]. Indeed, in mouse embryonic stem cells, the depletion of DNA (cytosine-5)-methyltransferases, Dnmt3α and Dnmt3β, increased centromeric SCEs [100]. This notion is also supported by the observation that defects in human methyltransferases, including DNMT3B, are linked to immunodeficiency-centromeric instability-facial anomalies (ICF) syndrome [114]. However, there is a growing recognition that functionally active CENP-A bound centromeric DNA is hypomethylated [52] (Figure 1), hence this model is unlikely to entirely explain the mechanism ensuring centromere integrity. Focusing on core centromeres, CENP-A and other CCAN constituents, CENP-C, CENP-T, and CENP-W, have been described to protect centromeres against extensive rearrangements, as discussed in section 2.2.2 [96], [112]. More recently, a member of the Sucrose Non-Fermenting 2 (SNF2) family of ATPases, ERCC6L2, has also emerged as a distinctive constitutive centromere-associated protein, which is linked to replication processivity at centromeres [115]. ERCC6L2 preserves levels of heterochromatic markers, H3K27me2, H3K27me3, and histone H1 at centromeres, fine-tuning the speed of DNA replication. Further, ERCC6L2 was found to limit DSB resection and maintain CENP-A, CENP-B, and CENP-C levels. Through these multifaceted mechanisms, ERCC6L2 is proposed to limit error-prone DNA repair at centromeres [115].

While budding yeast centromeres contain no repetitive sequences [1], studies using this model organism also reveal that centromeres harbour inherent properties directing them to undergo local recombination [116]. Specifically, cohesin-mediated centromeric DNA loops limit DNA resection and the subsequent homology search, such that recombination events are restricted to take place *in cis* [77]. In fission yeast, Rad51 is also found to suppress centromere-derived GCRs by facilitating non-crossover recombination between repeats on the same chromatid, otherwise inhibiting error-prone repair pathways such as SSA, BIR, or MMEJ, which occur with higher prevalence on chromosome arms [84], [101], [111]. As discussed in section 2.1, studies in mice and humans also indicate that centromere repeats are repaired *in cis* [72], [73]. These observations collectively support that, regardless of centromeric DNA sequences and their degree of repetitiveness, non-crossover recombination events commonly occur locally at these regions to maintain their integrity and limit GCRs resulting from aberrant repair events, although the level of precision during repair remains to be established.

### 2.3 Centromeres in mitosis

As cells enter mitosis, chromosomes undergo dynamic architectural changes, including condensin-mediated chromosome folding, the resolution of cohesion and global silencing of transcription, apart from centromeres. Also, the kinetochore machinery is stabilised and attached to spindle fibres in early mitosis. In late mitosis, chromosomes are segregated into daughter cells and decondense. Such changes provide opportunities and challenges for the stability of chromosomes and centromeres. Some unique features of centromeric chromatin in mitosis are overviewed below.

#### 2.3.1 dsDNA catenanes assisting sister chromatid cohesion

**Figure 5:**
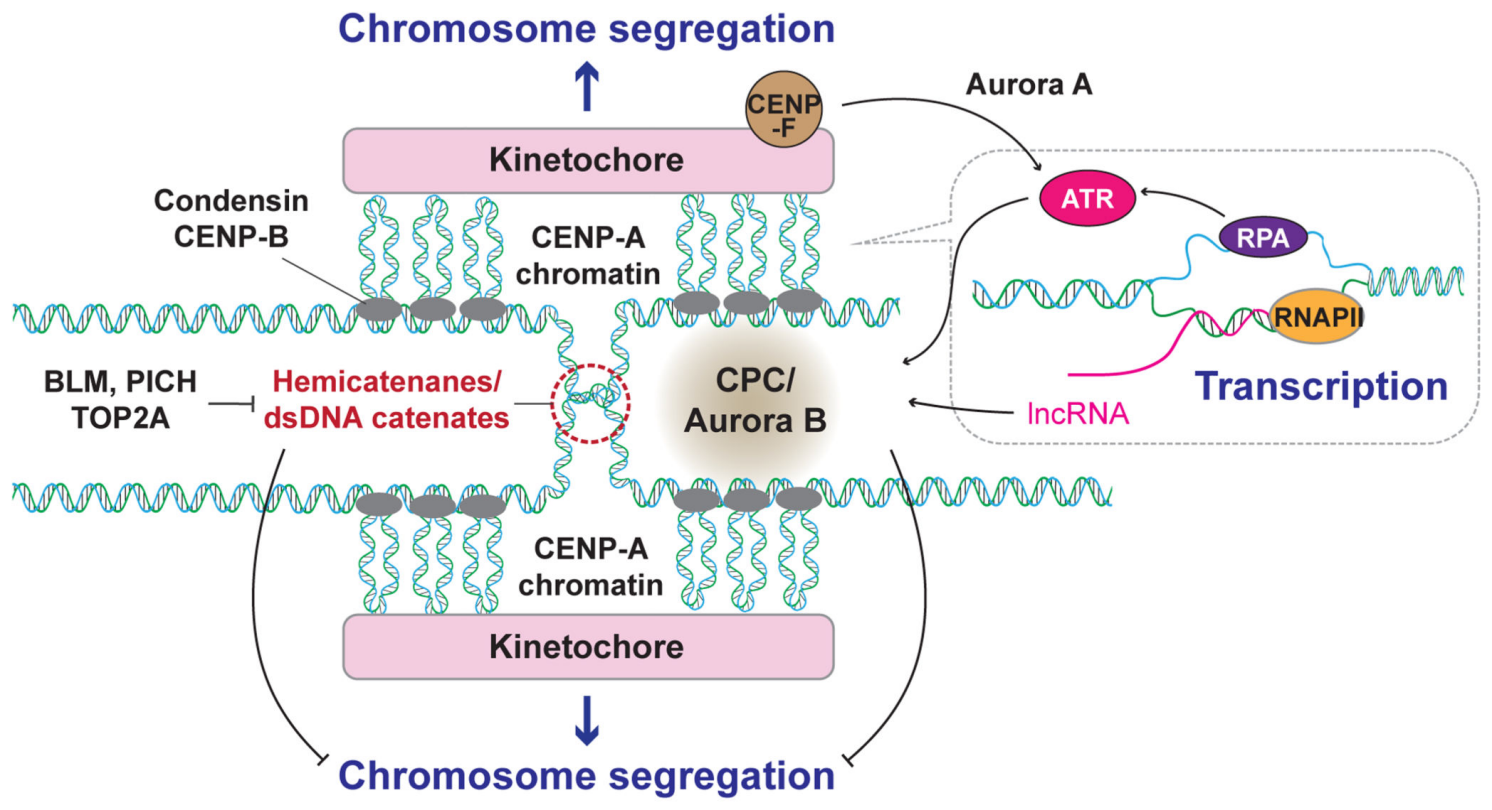
Events at centromeres in mitosis Centromeres are subjected to RNA polymerase II (RNAPII)-mediated transcription, which is important for recruiting the chromosome passenger complex (CPC) and maintaining the spindle assembly checkpoint (SAC). Displaced single stranded DNA is protected by replication protein A (RPA) binding. Centromeres are also enriched with double stranded DNA loops and catenates. The DNA loops are important to withstand the tension from microtubule pulling forces in mitosis. DNA catenates contribute to sister chromatid cohesion until they are resolved by topoisomerase IIα (TOP2A) in late anaphase; they block abrupt inactivation of the SAC at metaphase and ensure chromosome segregation can proceed.

In budding yeast, super-helical tension generated during replication termination is resolved by TOP2A, producing dsDNA catenanes, which are found highly enriched at centromeres [117]. In line with this observation, in many organisms, dsDNA catenanes are thought to play a role in centromere cohesion and are generally resolved before anaphase onset. Delayed TOP2A-mediated decatenation leads to the maintenance of catenanes into anaphase and formation of centromeric ultrafine anaphase bridges (C-UFBs) (Figure 5). UFBs are uncondensed, dechromatinised topological DNA entanglements between sister chromatids that cannot be detected using typical DNA dyes including DAPI [118], [119]. C-UFBs arise commonly in unperturbed cells [120], [121] and are resolved by the collective action of dsDNA translocase PICH [122], BLM [123], and TOP2A [124]. In short, PICH localises to stretched DNA in anaphase [125]–[127], recruiting BLM and TOP2A to remove distorted DNA structures [118], [126], [128], [129]. After TOP2A-mediated decatenation, the dsDNA forming C-UFBs can be fully disentangled, and chromatid separation and telophase can proceed.

Whether C-UFBs have a physiologically important role during mitosis remains contentious. Due to their high frequency, it has been suggested that C-UFBs may act to sense tension in anaphase to maintain centromere cohesion or satisfy the spindle assembly checkpoint [130], [131], and that complete centromere decatenation only occurs after anaphase onset [119], [121]. Alternatively, their presence could simply reflect impaired TOP2A decatenation activity at centromeres [131]. Nevertheless, it seems clear that timely C-UFB resolution and complete separation of nascent DNA strands prevents centromere instability and cell death [132]. Indeed, unresolved UFBs increase 53BP1 nuclear bodies, which mark broken DNA, and PICH depletion can give rise to mitotic defects such as micronuclei and aneuploidy. Therefore, uncontrolled UFB breaks can increase the risk of genome instability, potentially conferring altered centromere length and translocations which can contribute to changes in gene expression and cancer [3], [127], [133].

#### 2.3.2 R-loops and lncRNA mediating mitotic checkpoint signalling

RNAPII-mediated transcription, which is largely toxic in S phase, once again becomes essential in mitosis for promoting the functional kinetochore structure. Both *in vitro* and *in vivo*, centromere transcripts were shown to stabilise CENP-C and the chromosome passenger complex (CPC), a key regulatory complex comprising Aurora B kinase that controls chromosome segregation during mitosis. Inhibition of this process delays anaphase progression and produces lagging chromosomes [134], [135]. Furthermore, R-loop-dependent CPC recruitment to the mitotic centromere is found to maintain centromere cohesion, which is subsequently resolved by Aurora B kinase activity [136].

There is a separate ATR-mediated mechanism which is reported to support mitotic progression through R-loops (Figure 5). While ATR activity is suppressed on centromeric chromatin during S phase [54], [87] (discussed in section 2.2.1), it was found to have a vital role in unperturbed mitosis in chromosome segregation [55]. In mitosis, the displaced ssDNA of centromeric R-loops is bound by RPA, which in turn promotes recruitment of ATR [55]. ATR localisation is further compounded by kinetochore components, Aurora A and CENP-F, and, in prometaphase, ATR activates CHK1, which then promotes the recruitment and activation of Aurora B for chromosome segregation.

Collectively, these observations highlight the importance of centromere transcription and R-loops in unperturbed mitosis by facilitating Aurora B activation, without which, mitotic defects would negatively impact genome stability. This appears to be distinct from the harmful impact of R-loops in S phase (section 2.2.2), further supporting that centromeric transcription is a tightly regulated process to maintain centromere and genome stability.

#### 2.3.3 Chromatin architecture withstanding microtubule pulling force

Micronuclei containing centromeric DNA sequences resulting from mitotic spindle defects suggest that mechanical forces can result in centromere shearing in mitosis [137]. (Peri)centromeric DNA loops have been proposed to alleviate the impact of tension generated by spindle pulling forces, and in this way, promote proper chromosome segregation [138]. Centromeric DNA loops are generated by the ring-like structure SMC complexes and other centromere-associated factors, such as dimer-forming CENP-B. Chardon *et al*. utilised high resolution optical tweezers and atomic force microscopy to identify highly dynamic CENP-B-dependent DNA loops on centromeric chromatin (Figure 5) [139], approximately 350-500 base pairs in size, agreeing with previous EM observations of *in vitro* replicated BACs containing human α-satellite sequences (see section 2.2.1) [54]. CENP-B-dependent loops facilitate centromere flexibility to withstand DNA tension from microtubule pulling forces in mitosis and, in their absence, centromere fragility was increased and 53BP1 nuclear bodies were enriched in the following G1 [139].

A similar principle was also demonstrated by Jones *et al*. [140]. Here, PLK1 was found to inhibit BLM, which catalyses centromeric DNA unwinding to assist DNA integrity upon chromosome segregation at C-UFBs (see section 2.3.1). PLK1 inactivation, hence the premature activation of BLM-mediated DNA unwinding, triggers severe centromere decompaction, which destabilises spindle pulling forces, leading to rupture and whole chromosome arm splitting [140]. These observations further support the importance of DNA loops for centromere integrity in metaphase to prevent GCRs and fragmentation.

#### 2.3.4 Repair of centromeric DNA breaks in mitosis

It is largely believed that the repair of DNA strand breaks is suppressed during mitosis, except at a very early stage when MiDAS takes place in cells exposed to mild replication stress during interphase [141]. These cells were found to enter mitosis prematurely before completing DNA replication at ‘difficult-to-replicate’ regions such as common fragile sites and telomeres. In the early stages of chromosome condensation, those under-replicated regions are actively cleaved by endonucleases such as MUS81, providing an opportunity to complete DNA replication through a BIR-like mechanism [141]. Alternatively, such regions can continue DNA synthesis [142], [143], potentially assisted by the removal of replication roadblocks such as the transcription machinery. While direct evidence of MiDAS at centromeres has not been established, there are some indications that centromeres are affected. For example, our recent work found that the inhibition of RAD51 in mitosis reduced overall MiDAS events, increased aberrant centromeric SCEs and mitotic duration [143]. These observations indicate a mitotic role of RAD51 at centromeres, albeit not necessarily through its repair capacity. Of note, during the preparation of this manuscript, evidence emerged indicating that mitotic DNA break repair can take place in a manner dependent on POLQ [144], a key enzyme that catalyses MMEJ (discussed in section 2.2.3, Figure 4), [145], [146]. This observation may explain, at least in part, how centromeric DNA breaks in mitosis could result in SCEs in the absence of RAD51.

In a vast number of cases, cells appear to choose not to repair broken DNA but, if needed, to protect them until G1 to avoid incorrect repair resulting from rapid and extensive changes in the chromosome architecture during mitosis. Such protective roles have been reported for 53BP1 and TOPBP1 [147], [148], although it is unclear whether they also act at centromeres. TOP2A, the BLM dissolvasome complex and the SLX4 resolvase complex all exhibit the highest activity in mitosis. Even if these lesions are not immediately repaired, this could also imply a biological advantage to unusual mitotic DNA structures and breaks (as in section 2.3.1).

### 2.4 Centromeres during quiescence

Quiescence is a cellular state characterised by a temporary exit from the cell cycle while still retaining the ability to divide. In humans, there are many different types of quiescent cells, including somatic stem cells, lymphocytes, and oocytes, comprising considerable parts of the adult body [149]. These cells commonly exhibit the down-regulation of central cell cycle kinases and global silencing of transcription [149]. Intriguingly, however, centromeres appear to remain transcriptionally active, similar to mitosis and early G1. It was found that RNAPII-mediated centromere transcription during quiescence facilitates the gradual turnover of CENP-A at centromeres. RNAPII mediates the eviction of CENP-A nucleosomes, while HJURP mediates CENP-A re-deposition. When this mechanism is perturbed, quiescent cells progressively lose CENP-A and their ability to return to a proliferative state [150]. Beyond this finding, the centromere biology in quiescent cells remains largely unexplored, partly due to the lack of experimental tools and approaches to directly assess centromere-associated phenotypes.

#### 2.4.1 Repair of centromeric DNA breaks during quiescence

**Figure 6:**
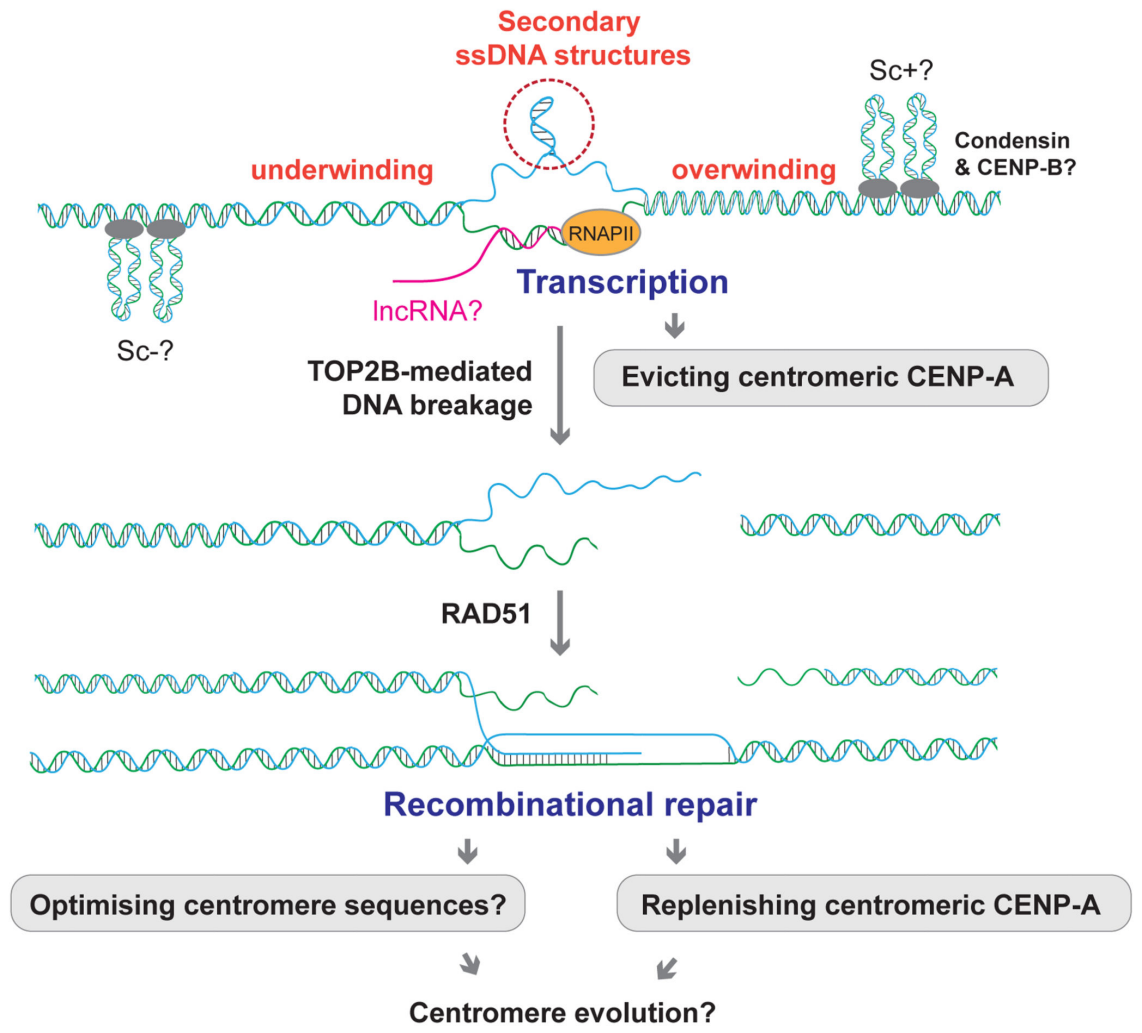
Events at centromeres during quiescence Centromeres are subjected to RNA polymerase II (RNAPII)-mediated transcription, which is important for dynamic turnover of CENP-A during quiescence. Accompanying DNA unwinding, torsional stress and RNA/DNA hybrid (R-loop) formation could trigger the creation of secondary DNA structures, potentially leading to the induction of DNA breaks. Topoisomerase IIβ (TOP2B) is experimentally shown to mediate the induction of DNA breaks, which are repaired by RAD51-mediated recombination. This is important to maintain the level of CENP-A at centromeres. Recombination in centromeres might also underlie the co-evolution of optimal centromere DNA sequences. Sc+ and Sc- denote positively or negatively supercoiled dsDNA, respectively.

We have recently developed a microscopy-based methodology to detect DNA breaks at repetitive regions and revealed a previously unrecognised characteristic of centromeres during quiescence. This methodology, exo-FISH, exploits *in vitro* end resection of undenatured DNA by exonuclease III, which digests DNA from 3’ ends, thus exposing ssDNA. The resulting ssDNA is subsequently visualised with a fluorescently labelled complementary probe (FISH). The FISH signal intensities were then used to infer the level of DNA damage. Through this new method, we observed an increase in spontaneous DNA breaks at centromeres in quiescent, non-cancerous human RPE1 cells. These breaks were newly introduced during quiescence and were dependent on the enzyme topoisomerase IIβ (TOP2B) (Figure 6) [51]. Further to this observation, we found a role for RAD51-mediated HR repair at these centromere breaks. RAD51’s strand exchange activity was important to limit the level of centromeric DNA breaks and RAD51 depletion led to the reduction of CENP-A at centromeres. CENP-A exchange in quiescent cells was accelerated between four and seven days after serum starvation in RPE1 cells [150], which is in line with *de novo* centromeric DNA break induction [51]. Therefore, active HR repair is likely important to maintain CENP-A occupancy in quiescent RPE1 cells, though further investigation is indispensable to fully understand the underlying molecular mechanism.

Our study focused on assessing centromeres in quiescent RPE1 cells, but it is plausible that centromere DNA breaks could be induced by other mechanisms in different cell types. For example, topoisomerase I (TOP1)-dependent processing of R-loops was found to drive SSB and DSB induction in non-cycling primary human lung embryonic WI38 fibroblasts [151]. Removal of TOP1 cleavage complexes (TOP1cc) by the tyrosyl-DNA-phosphodiesterase I (TDP1) and R-loop cleavage by endonucleases such as XPF, XPG, and FEN1 can induce staggered SSBs, effectively forming DSBs [151]. Although this was not shown at centromeres specifically, active centromere transcription during quiescence [150] could promote the accumulation of centromere breaks through a similar mechanism.

## Concluding remarks

Centromeric DNA is challenged across the cell cycle from numerous sources including RNAPII-dependent transcription, processing of secondary DNA structures, and topologically generated structures, including dsDNA catenates. The consequences of these features vary in how they promote and facilitate centromere function such as CENP-A deposition, or impede normal centromere processes like replication and contribute to DNA break generation. Recombination is now regarded as a common hallmark of centromeres, with *cis*-acting non-crossover resolution maintaining centromere stability whilst crossover resolution and other error-prone repair mechanisms are detrimental and can drive tumourigenesis through loss of α-satellite sequences and GCRs. With recent advancement in experimental approaches and technologies, such as exo-FISH and long-read DNA sequencing, we are moving into an exciting era for investigating centromeres in greater depth. Future work should address how the cellular response to centromere instability is regulated in different contexts to preserve centromere functionality during both growth and quiescence.

## Data Availability

No data was used for the research described in the article.
